# Clinical Efficiency of Vasopressin or Its Analogs in Comparison With Catecholamines Alone on Patients With Septic Shock: A Systematic Review and Meta-Analysis

**DOI:** 10.3389/fphar.2020.00563

**Published:** 2020-05-06

**Authors:** Ren-qi Yao, De-meng Xia, Li-xue Wang, Guo-sheng Wu, Yi-bing Zhu, Hong-qiang Zhao, Qi Liu, Zhao-fan Xia, Chao Ren, Yong-ming Yao

**Affiliations:** ^1^Trauma Research Center, Fourth Medical Center of the Chinese PLA General Hospital, Beijing, China; ^2^Department of Burn Surgery, Changhai Hospital, The Second Military Medical University, Shanghai, China; ^3^Department of Emergency, Changhai Hospital, The Second Military Medical University, Shanghai, China; ^4^Medical Research and Biometrics Center, Fuwai Hospital, National Center for Cardiovascular Diseases, Peking Union Medical College, Beijing, China

**Keywords:** vasopressin, terlipressin, selepressin, norepinephrine, septic shock

## Abstract

**Background:**

Vasopressin is an efficient remedy for septic shock patients as its great capacity in promoting hemodynamic stabilization. The aim of current systematic review and meta-analysis is to compare the clinical efficiency of vasopressin or its analogs with sole catecholamines on patients with septic shock.

**Methods:**

A systematic search of Cochrane Library, EMBASE, and PubMed online databases was performed up to 30 Oct 2019 to identify randomized controlled trials comparing use of vasopressin or its analogs (e.g., terlipressin, selepressin) with administration of catecholamines alone.

**Results:**

We included 23 RCTs with 4,225 patients in the current study. Compared with solely use of catecholamines, administration of vasopressin or its analogs was not associated with reduced 28-day or 30-day mortality among patients with septic shock [RR=0.94 (95% CI, 0.87–1.01), *P*=0.08, I^2^ = 0%]. The result of primary endpoint remained unchanged after conducting sensitivity analysis. Despite a significantly higher risk of digital ischemia in patients receiving vasopressin or its analogs [RR=2.65 (95% CI, 1.26–5.56), *P* < 0.01, I^2^ = 48%], there was no statistical significance in the pooled estimate for other secondary outcomes, including total adverse events, arrhythmia, acute myocardial infarction (AMI) and cardiac arrest, acute mesenteric ischemia, ICU/hospital length of stay, and mechanical ventilation (MV) duration.

**Conclusions:**

The administration of vasopressin or its analogs was not associated with reduced 28-day or 30-day mortality among patients with septic shock, while an increased incidence of digital ischemia should be noted in patients receiving agonists for vasopressin receptors.

## Introduction

Sepsis is a common yet complex disorder that remains one of the major causes of death among patients admitted to intensive care units (ICUs) (Hotchkiss et al., 2016; Singer et al., 2016). Septic shock, a severe subset of sepsis, represents a lethal yet intractable condition for ICU patients (Singer et al., 2016). The mortality rate shows significant increase after identification of septic shock, from 40% to 80% due to the untenable hemodynamic status and persistent low blood perfusion (Gaieski et al., 2013). Administration of norepinephrine is the first-line choice and an effective treatment for improving survival of septic shock patients (Rhodes et al., 2017). Furthermore other vasopressor medications, such as epinephrine, dopamine, and vasopressin and its analogs, also showed noteworthy benefits in ameliorating vascular resistance, achieving target mean arterial pressure (MAP) levels, and further maintaining efficient perfusion in tissues and crucial organs during septic shock (Vincent and De Backer, 2013).

However, patients with septic shock are prone to become insensitive to catecholamines, and even develop vasopressin deficiency (Russell, 2007). Meanwhile, growing evidences have shown that catecholamine associated adverse effects might be unavoidable by exclusively using catecholamines, including myocardial ischemia and tachycardia, which also poses a great threat to the prognosis of septic shock patients (Dunser and Hasibeder, 2009; Levy et al., 2010; Schmittinger et al., 2012; Vincent and De Backer, 2013). Given that, several studies have demonstrated that combined administration of vasopressin or its analogs with catecholamines not only reduced the use of catecholamines, but also potentially attenuated catecholamine associated adverse effects (De Backer et al., 2010; Hammond et al., 2018). Thus, administration of vasopressin is recommended by recently issued Surviving Sepsis Guidelines to get the target MAP level, along with benefit of reducing NE dosage (Rhodes et al., 2017). Likewise, analogs of vasopressin, including terlipressin and selepressin, were reportedly beneficial for improving hemodynamic status, attenuating sepsis related vasodilatation and tissue edema, which were largely attributed to its selective stimulation of V_1_ receptors (Rehberg et al., 2010; Laporte et al., 2011). Recently, results from two large randomized controlled trials (RCTs) were published and aroused the usage and effects of vasopressin or its analogs on the prognosis of septic shock patients (Liu et al., 2018; Laterre et al., 2019). Of note, several well-designed systematic review and meta-analyses have evaluated the effects and safety of vasopressin or its analogs on the occurrence of adverse effects and short-term mortality, while the conclusions of those studies were divergent from each other (Serpa Neto et al., 2012; Avni et al., 2015; McIntyre et al., 2018; Jiang et al., 2019; Nagendran et al., 2019). This might be due to disparate inclusion criteria and methodologic selection.

Therefore, we plan to conduct an updated systematic review and meta-analysis of RCTs to test the clinical efficiency of vasopressin or its analogs versus catecholamines alone on patients-centered outcomes during septic shock. Furthermore, we aim to explore if selective V_1_ receptor agonists (terlipressin, selepressin) show beneficial effects on clinical outcomes of septic shock patients.

## Materials and Methods

**Figure 1:**
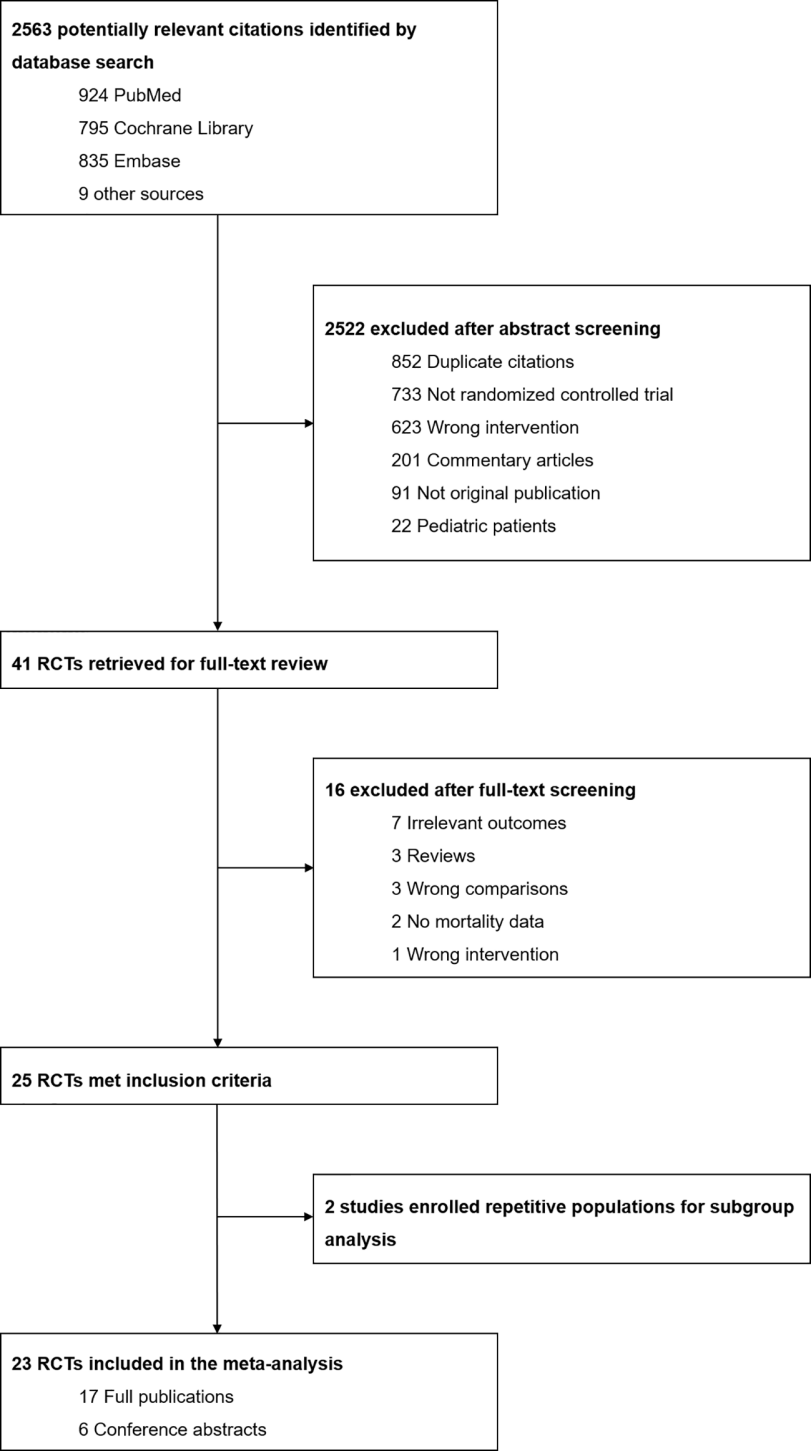
Flowchart for selection process.

The current systematic review and meta-analysis was performed in accordance with the Preferred Reporting Items for Systematic Reviews and Meta-Analysis (PRISMA) statements (Moher et al., 2009) (see **Figure 1**).

### Eligibility Criteria and Outcome Measurement

RCTs comparing the use of vasopressin or its analogs (e.g. terlipressin, selepressin) with catecholamines alone (e.g. norepinephrine, dopamine) or placebo among adult patients with septic shock were included in the current study.

The primary endpoint was 28-day or 30-day mortality, which was preferentially reported by majority of included trials. In case of unreported 28-day or 30-day mortality, we contacted the authors for inquiring the original data or considered the closest available mortality data. Secondary outcomes were listed as follows: total adverse events, arrhythmia, acute myocardial infarction (AMI) and cardiac arrest, acute mesenteric ischemia, digital ischemia, ICU length of stay, hospital length of stay and duration of mechanical ventilation. For studies reported length of stay in median and interquartile range, we converted those data into mean and standard deviation, respectively, by using algorithm provided by statisticians.

### Literature Search

A systematic search was conducted by applying multiple online databases, including Cochrane Library, EMBASE, and PubMed. Relevant studies up to 30 Oct, 2019 were reviewed irrespective of languages and publication types. We conceived search strategies that involved following Medical Subject Heading (MeSH) terms: “Sepsis,” “Vasopressins,” “Arginine Vasopressin,” “Deamino Arginine Vasopressin,” “Lypressin,” “Felypressin,” “Ornipressin,” and “terlipressin.” Detailed search strategies were summarized in **Supplemental File 1**. Besides, unpublished trials and conference abstracts were hand-searched by the authors to obtain additional studies. We also identified references through screening the reference lists of eligible reviews and trial registries.

### Study Selection and Data Collection

Two reviewers independently screened the titles and abstracts of relevant studies for enrolling eligible trials. If the abstract of a potentially eligible trial failed to provide sufficient information, the full paper was subsequently obtained to determine its eligibility. In both inclusion and exclusion processes, divergent opinions between the two authors were resolved by discussion. Otherwise, a consulting group that consisted of several experts was involved in when a consensus could not be reached.

Independently, two reviewers extracted data from included studies with a predesigned sheet. The primary and secondary outcomes were obtained across all eligible trials. In addition, detailed information about studies and participants’ characteristics were recorded accordingly, including year of publication, first author, study design, total number of enrollments, type of intervention, demographic characteristics, clinical settings and complications. Similarly, the inconsistency of extracted data and disagreement were resolved by discussion.

### Assessment of Risk of Bias

The methodological quality of all included studies was assessed by using the Jadad scoring system, which was comprised of three dimensions (randomization, double-blinding as well as withdrawals and dropouts). Each trial was assigned a score of 0 to 5, a study with score higher than 3 indicated high quality and low risk of bias, or else, revealing high risk of bias.

### Data Synthesis and Analysis

We applied risk ratios (RRs) for dichotomous outcomes, while mean differences (MDs) were used for pooling continuous data. The pooled results were calculated with 95% confidence intervals (CIs). Methodological heterogeneity of each outcome was measured by using both χ^2^ test and I^2^ statistics. In either case, I^2^ > 50% or P value < 0.10 (χ^2^ test) was regard as significant heterogeneity. We applied random effects model combined with Mantel-Haenszel method for certain outcomes when statistical heterogeneity existed, or else, fixed effects model was used. A two-sided P value < 0.05 was deemed as statistically significance. To measure publication bias across studies, the funnel plot of the primary endpoint was constructed and visually inspected by authors. Additionally, we performed Harbord and Peter tests to further evaluate the publication bias.

Subgroup analysis combined with sensitivity analysis were performed to test the robustness and consistency of our primary endpoint, as well as finding potential influencing factors. We stratified all included RCTs by administration of vasoactive agents (vasopressin or it’s analogs), clinical settings, outcome report, risk of bias, as well as publication types (full text or abstract).

All statistical analyses were performed using R software (version 3.6.1).

### Evaluation of the Quality of Evidence

The quality of evidence of each outcome was evaluated in line with the Grading of Recommendations, Assessment, Development and Evaluation (GRADE) criteria (Guyatt et al., 2008). This procedure was conducted with GRADE Pro software 3.6 (McMaster University 2014, Hamilton, Canada).

## Results

### Trial Selection Processes and Characteristics of Included Studies

The current meta-analysis identified 2,563 relevant citations through searching online databases. After removing duplicates and subsequent screening of titles and abstracts, the full-text articles of 41 trials were reviewed, and 23 RCTs finally met the eligibility criteria (see **Figure 1**).

Twenty-three RCTs with 4,225 septic shock patients were enrolled in the systematic review and meta-analysis (Albanese et al., 2005; Lauzier et al., 2006; Morelli et al., 2008; Russell et al., 2008; Acevedo et al., 2009; Morelli et al., 2009; Han et al., 2012; Svoboda et al., 2012; Fonseca-Ruiz et al., 2013; Hua et al., 2013; Oliveira et al., 2014; Zambolim et al., 2014; Barzegar et al., 2016; Clem et al., 2016; Gordon et al., 2016; Xiao et al., 2016; Capoletto et al., 2017; Chen et al., 2017; Choudhury et al., 2017; Prakash et al., 2017; Russell et al., 2017; Liu et al., 2018; Laterre et al., 2019). The majority of enrolled trials were designed as single-center studies, while seven of them were multicenter studies. The administration of vasopressin was applied by 10 trials, and another 11 RCTs using terlipressin as the intervention, whereas selepressin was studied in two trials. Among them, six studies were published in conference abstract (Acevedo et al., 2009; Oliveira et al., 2014; Zambolim et al., 2014; Clem et al., 2016; Capoletto et al., 2017; Prakash et al., 2017). The detailed characteristics of all included RCTs were presented in **Table 1**.

**Table 1:** Characteristics of included randomized clinical trials.

Study	Design	No. of patients	Clinical settings	Intervention		Outcome measurement	Jadad score
				Treatment group	Comparison group		
Acevedo et al., 2009^*^	Single center	24	Cirrhotic patients with septic shock	Terlipressin	Alpha-adrenergic drugs	Hospital mortality ICU mortality	1
Albanese et al., 2005	Single center	20	Septic shock	Terlipressin	NE	Hospital mortality	2
Barzegar et al., 2016	Single center	30	Septic shock	Vasopressin plus NE	NE	28-day mortality ICU mortality	4
Capoletto et al., 2017	NA	250	Cancer patients with septic shock	Vasopressin	NE	28-day mortality 90-day mortality	3
Chen et al., 2017	Single center	57	Septic shock patients with ARDS	Terlipressin plus NE	NE	28-day mortality	3
Choudhury et al., 2017	Single center	84	Liver cirrhosis with septic shock	Terlipressin	NE	28-day mortality 48-h mortality	4
Clem et al., 2016^*^	Single center	82	Septic shock	Vasopressin plus NE	NE	28-day mortality	1
Fonseca-Ruiz et al., 2013	Single center	30	Septic shock	Vasopressin plus NE	NE	28-day mortality	4
Gordon et al., 2016	Multicenter	409	Septic shock	Vasopressin	NE	28-day mortality Hospital mortality ICU mortality	5
Han et al., 2012	Multicenter	139	Septic shock	Pituitrin	Dopamine or NE	28-day mortality	1
Hua et al., 2013	Single center	32	Septic shock patients with ARDS	Terlipressin	Dopamine	28-day mortality	2
Laterre et al., 2019	Multicenter	828	Septic shock	Selepressin	Placebo	30-day mortality 180-day mortality	5
Lauzier et al., 2006	Multicenter	23	Septic shock	AVP	NE	ICU mortality	3
Liu et al., 2018	Multicenter	526	Septic shock	Terlipressin	NE	28-day mortality	5
Morelli et al., 2008 ^a^	Single center	59	Septic shock	Terlipressin plus NE Terlipressin plus NE and dobutamine	NE	ICU mortality	3
Morelli et al., 2009 ^a^	Single center	45	Septic shock	Terlipressin plus NE AVP plus NE	NE	ICU mortality	3
Oliveira et al., 2014^*^	Single center	387	Septic shock	Vasopressin plus NE	NE	28-day mortality	3
Prakash et al., 2017^*^	Single center	184	Cirrhosis with septic shock.	Terlipressin plus NE	NE	30-day mortality	1
Russell et al., 2008	Multicenter	799	Septic shock	Vasopressin	NE	28-day mortality 90-day mortality	5
Russell et al., 2017	Multicenter	48	Septic shock	Selepressin	Placebo	28-day mortality	5
Svoboda et al., 2012	Single center	30	Septic shock	Terlipressin plus NE	NE	28-day mortality	3
Xiao et al., 2016	Single center	32	Septic shock	Terlipressin plus NE	NE	7-day mortality 24-h mortality	3
Zambolim et al., 2014^*^	Single center	107	Septic shock and cancer	Vasopressin	NE	28-day mortality	1

NE, norepinephrine; AVP, arginine vasopressin; ICU, intensive care unit; ARDS, acute respiratory distress syndrome.

NA, not available from article or author.

*Published in abstract only.

a Morelli et al., 2008 and Morelli et al., 2009 have compared three arms.

### Risk of Bias Assessment

Each RCT in our study was assigned a score of 0 to 5 by using Jadad scoring system (**Supplemental Table S1**). The majority of enrolled trials met the randomization requirements by using distribution methods. Among them, 8 RCTs (34.8%) had Jadad score of 4 or 5, while eight studies (34.8%) scored 3, indicating that 16 included RCTs (69.6%) were of low risk of bias. While seven studies (30.4%) were given Jadad score of 1 or 2, indicating a high risk of bias that was mainly due to the publication types and lack of blinding.

### Primary Outcome: 28-Day or 30-Day Mortality

**Figure 2:**
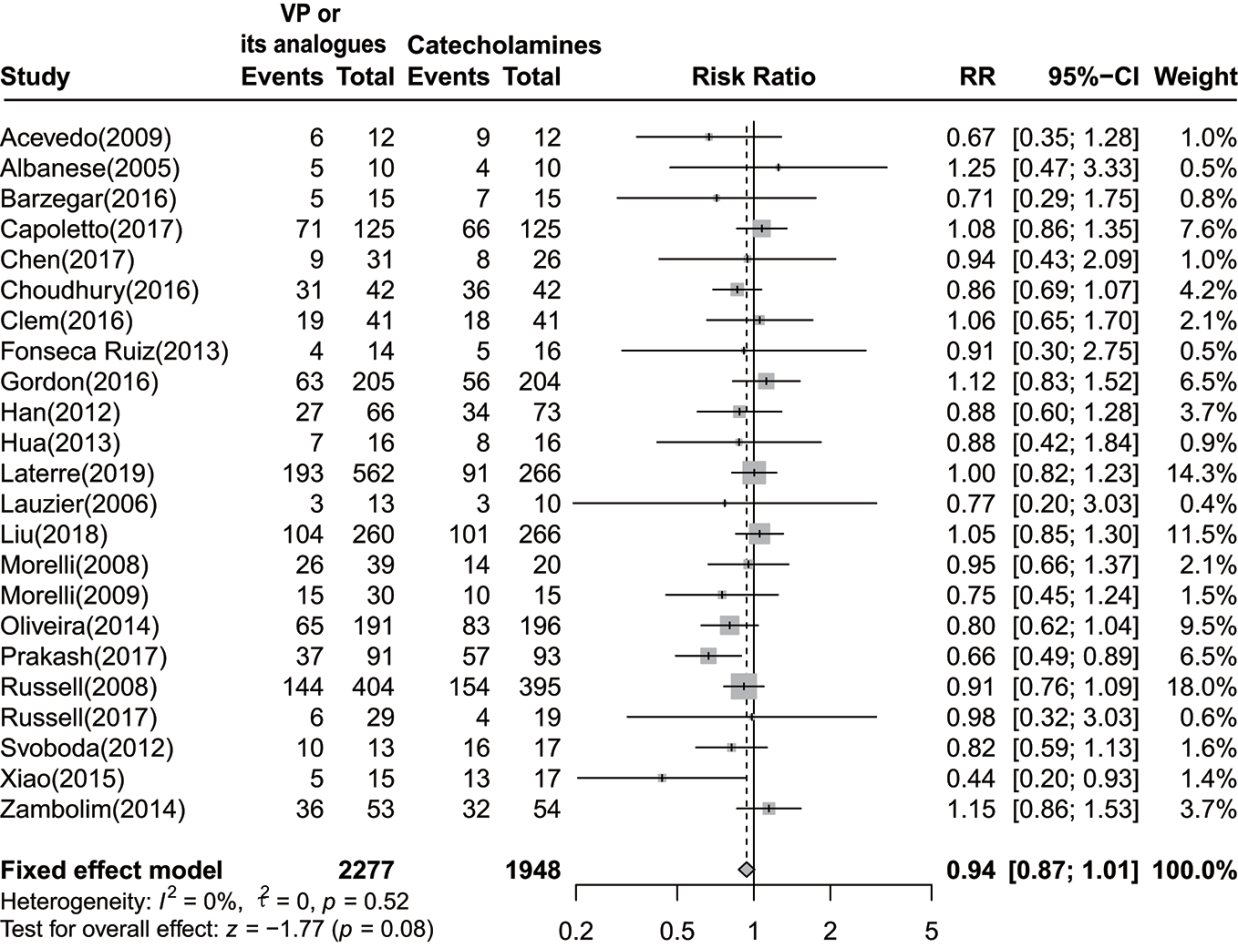
Forest plot of 28-day or 30-day mortality comparing vasopressin or its analogs to catecholamines alone among septic shock patients. VP, vasopressin; RR, risk ratio; CI, confidence intervals.

Mortality data were accessible in 23 trials, 17 of them have reported 28-day or 30-day mortality, while six studies reported other endpoints. Pooled data form 23 trials demonstrated no significant reduction in 28-day or 30-day mortality among patients who were given vasopressin or its analogs when comparing to those using catecholamines alone [RR=0.94 (95% CI, 0.87–1.01), *P*=0.08, I^2^ = 0%]. The forest plot was shown in **Figure 2**.

As summarized in **Table 2**, the consequence remained unchanged when conducting sensitivity analysis. As stratifying by interventions, RR of the vasopressin group was 0.96 [(95% CI, 0.87–1.06), *P*=0.44, I^2^ = 0%]. For studies incorporated septic shock patients without other primary complications, the combined RR was 0.94 [(95% CI, 0.87–1.03), *P*=0.20, I^2^ = 0%]. After removing trials reported mortality in other phases, there was no significant difference between two groups [RR=0.95 (95% CI, 0.88–1.02), *P*=0.18, I^2^ = 0%]. Besides, combined RR of pooling data from studies with low risk of bias was 0.95 [(95% CI, 0.88–1.03), *P*=0.22, I^2^ = 0%]. Of note, combined RR for studies published in full text and abstract were 0.95 [(95% CI, 0.87–1.04), *P*=0.30, I^2^ = 0%], and 0.90 [(95% CI, 0.79–1.02), *P*=0.09, I^2^ = 55%], respectively.

**Table 2:** Subgroup analysis and sensitivity analyses on primary outcome.

Subgroup		No. of studies	No. of patients	RR (95% CI)	I^2^	*P* value
**Intervention**						
	Vasopressin	10	2,147	0.96 (0.87–1.06)	0%	0.44
	Analogs	13	2,093	0.91 (0.82–1.01)	3%	0.07
**Clinical setting**						
	Septic shock only	16	3,487	0.94 (0.87–1.03)	0%	0.20
	Septic shock with complications	7	738	0.91 (0.80–1.03)	40%	0.15
**Outcome measurement**						
	28-day or 30-day mortality	17	4,022	0.95 (0.88–1.02)	0%	0.18
	ICU mortality	4	151	0.82 (0.63–1.07)	2%	0.15
**Risk of bias**						
	Low risk	16	3,637	0.95 (0.88–1.03)	0%	0.22
	High risk	7	588	0.87 (0.74–1.02)	29%	0.09
**Publication type**						
	Full text	17	3,191	0.95 (0.87–1.04)	0%	0.30
	Abstract	6	1,034	0.90 (0.79–1.02)	55%	0.09

ICU, intensive care unit; RR, risk ratio; CI, confidence interval.

**Figure 3:**
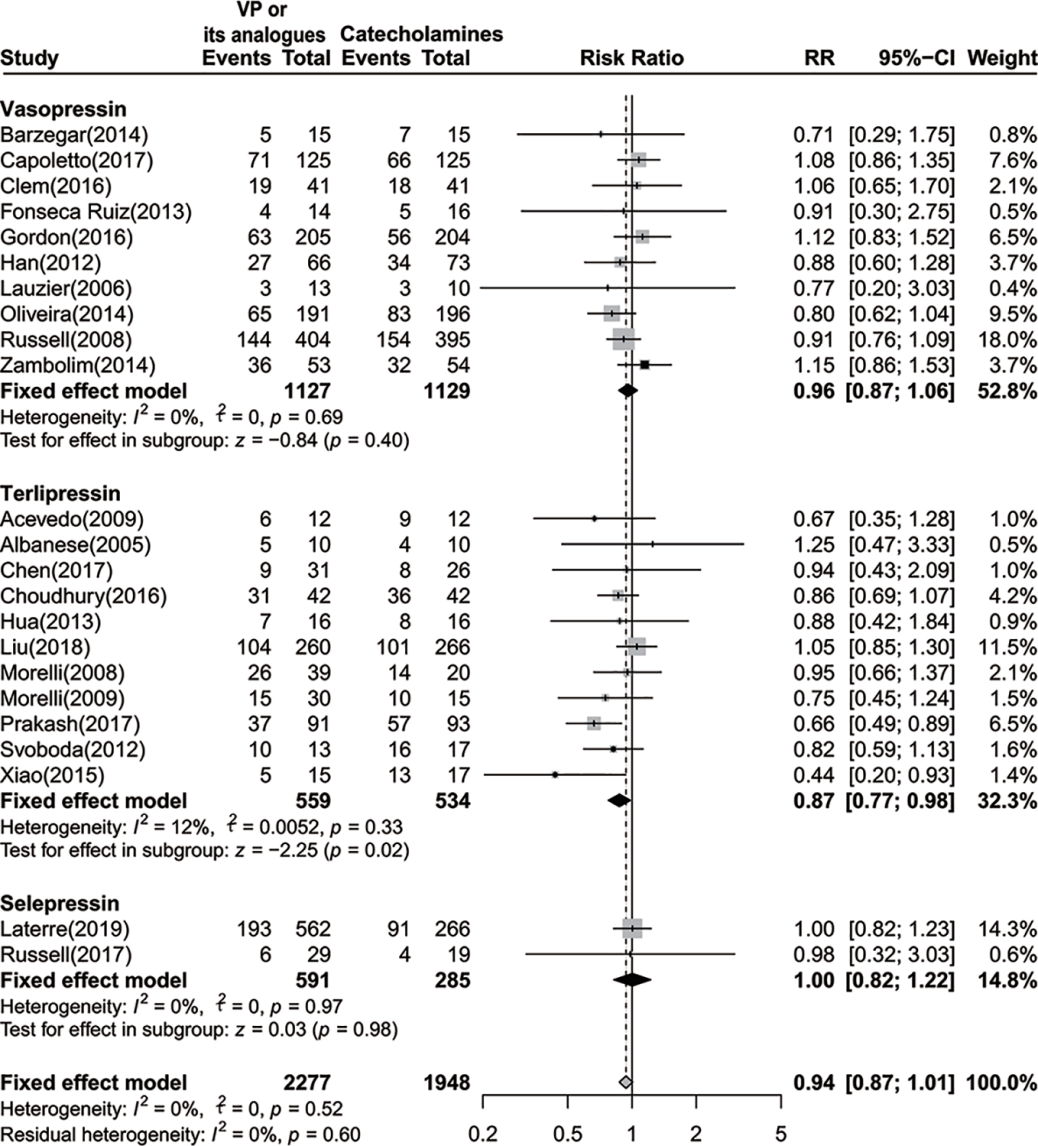
Forest plot of 28-day or 30-day mortality comparing vasopressin or its analogs to catecholamines alone stratified by disparate vasopressors. VP, vasopressin; RR, risk ratio; CI, confidence intervals.

Meanwhile, we performed a subgroup analysis based on disparate administration of vasoactive agents. As no statistical significances were observed in both vasopressin and selepressin subgroups, we found a significant lower 28-day or 30-day mortality rate among septic shock patients who received terlipressin [RR=0.87 (95% CI, 0.77–0.98), *P*=0.02, I^2^ = 12%] (**Figure 3**).

### Secondary Outcomes

#### Total Adverse Events

Given that 14 trials with a total of 3,206 patients reported total adverse events (980 events) (Lauzier et al., 2006; Russell et al., 2008; Morelli et al., 2009; Fonseca-Ruiz et al., 2013; Zambolim et al., 2014; Barzegar et al., 2016; Clem et al., 2016; Gordon et al., 2016; Xiao et al., 2016; Choudhury et al., 2017; Prakash et al., 2017; Russell et al., 2017; Liu et al., 2018; Laterre et al., 2019), administration of vasopressin or its analogs showed no significant association with the incidence of adverse effects [RR=1.21 (95% CI, 0.88–1.68), *P*=0.24, I^2^ = 73%] (**Supplemental Figure S1**). Although this outcome displayed a relatively high heterogeneity, the conclusion was proven to be stable after performing sensitivity analysis *via* excluding each study one at a time from the pooled estimate. Meanwhile, we found that study by Liu et al. was the main source of heterogeneity, which resulted in remarkable reduction in heterogeneity after exclusion (I^2^ from 73% to 41%).

#### Arrhythmia

The development of arrhythmia was documented in nine RCTs with 358 events (Russell et al., 2008; Morelli et al., 2009; Barzegar et al., 2016; Clem et al., 2016; Gordon et al., 2016; Choudhury et al., 2017; Russell et al., 2017; Liu et al., 2018; Laterre et al., 2019). We observed no significant difference in the occurrence of arrhythmia between two groups [RR=1.05 (95% CI, 0.87–1.27), *P*=0.61, I^2^ = 25%] (**Supplemental Figure S2**). Of note, trials by Laterre and colleagues accounted for over 76.4% weight. Even so, the conclusion was not altered after removing this study.

#### Digital Ischemia

**Figure 4:**
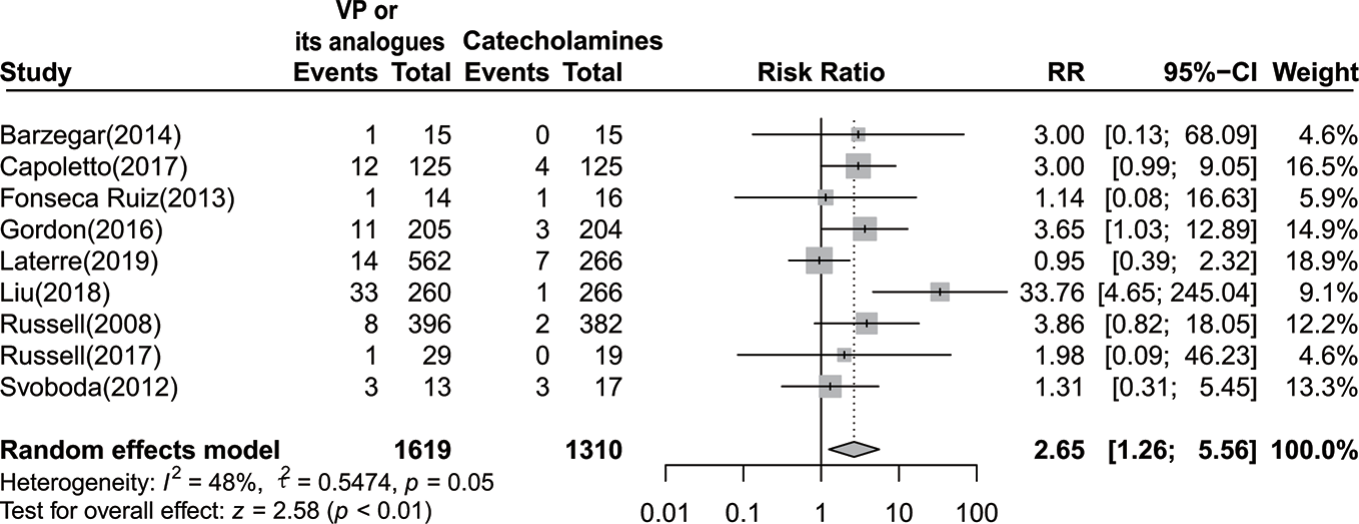
Forest plot of digital ischemia comparing vasopressin or its analogs to catecholamines alone among septic shock patients. VP, vasopressin; RR, risk ratio; CI, confidence interval.

By pooling data from nine studies with 2,929 participants (a total of 105 events) (Russell et al., 2008; Svoboda et al., 2012; Fonseca-Ruiz et al., 2013; Barzegar et al., 2016; Gordon et al., 2016; Capoletto et al., 2017; Russell et al., 2017; Liu et al., 2018; Laterre et al., 2019), we demonstrated that vasopressin or its analogs could lead to approximately two times risk of digital ischemia when compared to sole use of catecholamine in septic shock patients [RR=2.65 (95% CI, 1.26–5.56), *P* < 0.01, I^2^ = 48%] (**Figure 4**). We further confirmed that the result was robust through implementing sensitivity analysis, and excluding trial by Liu et al. could completely eliminate the heterogeneity (I^2^=0%).

#### ICU Length of Stay

Twelve included RCTs with 3,203 patients were eligible for the analysis of ICU length of stay (Morelli et al., 2008; Russell et al., 2008; Morelli et al., 2009; Han et al., 2012; Hua et al., 2013; Barzegar et al., 2016; Gordon et al., 2016; Capoletto et al., 2017; Chen et al., 2017; Choudhury et al., 2017; Liu et al., 2018; Laterre et al., 2019). Pooled effects revealed that administration of vasopressin or its analogs did not impact the length of stay in ICU [MD=–0.21 (95% CI, −0.75–0.33), *P* =0.44, I^2^ = 0%] (**Supplemental Figure S3**).

### AMI and Cardiac Arrest, Acute Mesenteric Ischemia, Hospital Length of Stay and MV Duration

The occurrence of AMI and cardiac arrest, acute mesenteric ischemia, and hospital length of stay as well as MV duration were reported in 7, 5, 6, 6 studies, respectively. None of those outcomes showed significant differences between two groups (**Supplemental Figure S4****–****S7**). Accordingly, the pooled effects and detailed information for those outcomes were presented in **Table 3**.

**Table 3:** Summary of primary and secondary outcomes.

Outcome		No. of events/No. of total patients		RR (95%CI)	MD (95%CI)	I^2^	*P* value	Quality of evidence^a^
		Vasopressin or Vasopressin’s analogs	Catecholamines alone					
**Primary endpoint**								
	28-day or 30-day mortality	891/2,277	829/1,948	0.94 (0.87–1.01)		0%	0.08	Moderate (Inconsistency)
**Secondary endpoint**								
	Total adverse effects	639/1,766	341/1,440	1.21 (0.88–1.68)		73%	0.24	Low (Inconsistency, Risk of bias)
	Arrhythmia	237/1,580	121/1,250	1.05 (0.87–1.27)		25%	0.61	Moderate (Inconsistency)
	AMI and cardiac arrest	69/1,480	43/1,162	1.05 (0.72–1.54)		0%	0.79	Moderate (Imprecision)
	Digital ischemia	84/1,619	21/1,310	2.65 (1.26–5.56)		48%	<0.01	Moderate (Inconsistency)
	Acute mesenteric ischemia	37/1,452	27/1,137	0.99 (0.60–1.63)		0%	0.96	Moderate (Inconsistency)
	ICU length of stay				−0.21 (−0.75–0.33)	0%	0.44	Low (Inconsistency, Indirectness)
	Hospital length of stay				0.15 (−1.39–1.70)	0%	0.85	Low (Inconsistency, Indirectness)
	MV duration				−0.47 (−1.18–0.24)	46%	0.19	Low (Indirectness, Imprecision)

ICU, intensive care unit; MV, mechanical ventilation; RR, risk ratio; MD, mean difference; CI, confidence interval.

a Quality of evidence of each outcome was assessed by using GRADE method.

### Quality of Evidence

The results of GRADE evaluation for all outcomes were summarized in **Table 3** and **Supplemental Table S2**. The quality of evidence for the primary endpoint and several secondary outcomes were ranked as moderate, including arrhythmia, AMI and cardiac arrest, digital ischemia, and acute mesenteric ischemia. Whereas, total adverse effects, hospital/ICU lengths of stay as well as MV duration were assessed as outcomes with low quality of evidence.

### Publication Bias

Publication bias was evaluated through visually inspecting the funnel plot, which revealed no evidence of publication bias. Furthermore, we applied Harbord test (*P*=0.47), and Peters test (*P*=0.25) to validate the funnel plot symmetry, which were both noted with no statistical significance (**Supplemental Figure S8**).

## Discussion

In the current systematic review and meta-analysis, we have evaluated the clinical efficiency of vasopressin or its analogs among patients with septic shock. We found that administration of vasopressin or selective V_1_ receptor agonists showed no benefits in reducing 28-day or 30-day mortality when compared with solely using NE, which was further validated by sensitivity analysis. Intriguingly, terlipressin might have potential prosurvival effects on septic shock patients independent of NE administration, as reported in subgroup analysis. Further large RCTs were required to strengthen the clinical efficiency of terlipressin among patients with septic shock. Meanwhile, no significant differences were observed in the incidence of most adverse effects between two groups, including total adverse events, arrhythmia, AMI and cardiac arrest, acute mesenteric ischemia. Of note, the occurrence of digital ischemia was more frequent among patients who received vasopressin or its analogs than those solely using catecholamines. Additionally, there were no significant improvements on ICU or hospital length of stay, as well as MV duration after administration of vasopressin or its analogs.

NE has long been recommended as the first-line vasoactive agent among patients with septic shock (Rhodes et al., 2017), and was broadly applied in restoring blood perfusion in tissue and vital organs and enhancing efficacy of fluid resuscitation (Avni et al., 2015; Hessler et al., 2016; Rhodes et al., 2017). However, high dose of NE reportedly caused severe side-effects, such as myocardial injury and immunological dysfunction, which made its long-lasting usage impossible (Andreis and Singer, 2016; Ukor and Walley, 2019). Vasopressin, an alternative non-catecholamine agent, was capable of increasing vascular resistance and restoring blood pressure *via* promoting arterioles constriction, which was also in favor of reducing the requirement of catecholamines among septic shock patients (Dunser et al., 2002; Holmes and Walley, 2004; McIntyre et al., 2018; Ukor and Walley, 2019). Meanwhile, it has been manifested that vasopressin could promote water resorption in renal tubules through its affinity to V_2_ receptors, thereby maintaining circulation blood volume (Ukor and Walley, 2019). Given that, recently issued Surviving Sepsis Guidelines has recommended adding vasopressin as the second-line vasoactive agent for the treatment of septic shock patients (Rhodes et al., 2017). Up to now, the Vasopressin or Septic Shock Trial (VASST) that was carried out by Russell and colleagues was the largest RCT for addressing this topic (Russell et al., 2008). However, they failed to reveal a reduced 28-day mortality among septic shock patients receiving NE combined with low-dose vasopressin (0.01 to 0.03 U/min) compared to those with sole use of NE, while a higher survival rate for vasopressin plus NE group was observed merely in the subset of patients with less severe septic shock (Russell et al., 2008). Meanwhile, other researchers have reported that administration of vasopressin in relatively late phases (after 12 h) and at high lactate levels might result in low response to vasopressin (Sacha et al., 2018). Additionally, growing evidences also suggested that vasopressin might be associated with decreased urine and cardiac outputs, as well as prothrombotic state due to its nonselective effects on V_1_ and V_2_ receptors (Torgersen et al., 2010; Salazar et al., 2015; Zhu et al., 2019). Therefore, the effects and safety of vasopressin should be taken cautiously.

In the current meta-analysis, we found that the use of vasopressin or its analogs had no effect on reducing 28-day or 30-day mortality among patients with septic shock. Whereas, it was disparate from many previous published systematic review and meta-analyses (McIntyre et al., 2018; Jiang et al., 2019). A recent study by Jiang et al. has concluded that the use of vasopressin might lead to reduced mortality among patients with septic shock (Jiang et al., 2019). Although the study was well-performed and comprehensive, some flaws might interfere the interpretation of their findings. They incorporated Malay’s study in the analysis of 28-day or 30-day mortality, but that study merely reported 24-h mortality, which might render an unsolvable bias. Besides, they failed to conduct sensitivity analysis for primary endpoint which was clearly unstable. Neto et al. performed a systematic review and meta-analysis by enrolling nine RCTs and demonstrated a reduced all-cause mortality in patients with vasodilatory shock, which showed statistical significance in septic shock subgroup (Serpa Neto et al., 2012). Of note, an updated study by McIntyre et al. showed that vasopressin in addition to catecholamines was associated lower mortality rate and incidence of atrial fibrillation among patients with distributive shock (McIntyre et al., 2018). The reason for the divergent results was mainly due to stricter inclusion criteria and incorporation of newly published RCTs. However, the current meta-analysis initially revealed a reduced 28-day or 30-day mortality among patients receiving terlipressin compared to those with catecholamines alone, which haven’t been reported by any other existing systematic review and meta-analyses.

Terlipressin, which is a synthetic long-acting analog of vasopressin, has high affinity toward V_1_ receptors on vascular smooth muscle (Leone et al., 2004). Theoretically, the use of terlipressin may result in less adverse effects than vasopressin does in treating septic shock, including thrombocytopenia, decreased cardiac output and hyponatremia (Rehberg et al., 2010; Salazar et al., 2015). Indeed, several pre-clinical studies have demonstrated that administration of terlipressin could reduce the requirement of NE, restore hemodynamic status and promote creatinine clearance among septic shock patients (Morelli et al., 2008; Morelli et al., 2009; Liu et al., 2018). Previous studies reported that terlipressin could improve renal function in patients diagnosed with hepatorenal syndrome, two trials further identified a reduced 28-day or 30-day mortality among septi shock patients complicated with liver cirrhosis after administration of terlipressin. Their findings were in accordance with the results of our analysis (Choudhury et al., 2017; Prakash et al., 2017). However, study by Zhu et al. didn’t reveal any pro-survival benefits of terlipressin among septic shock patients compared with sole administration of catecholamines (Zhu et al., 2019). They enrolled a total of 10 studies in their analysis, but one trial was conducted in pediatric ICU, which was largely disparate from other included trials and might potentially introduce bias. Besides, study by Liu et al. took over more than half of the total sample size, which might lead to significant publication bias and impaired robustness.

Selepressin is a novel vasopressin analog, which selectively stimulates V_1a_ receptors (Laporte et al., 2011; Russell et al., 2017). As demonstrated in pre-clinical studies, selepressin was capable of reducing fluid requirements and attenuating edema (Maybauer et al., 2014; He et al., 2016; Saad and Maybauer, 2017). In a recently published phase 2b/3 trial in septic shock patients, researchers didn’t find any favorable improvements in vasopressor- and ventilator-free days as well as 30-day mortality after administration of selepressin (Laterre et al., 2019).

As for secondary endpoints of interest, we found that the incidence of digital ischemia was significantly higher in patients receiving vasopressin or its analogs when compared to those solely using catecholamines. This finding was in line with many other systematic review and meta-analyses, which could be partially explained by vasopressin related cardiac output reduction and prothrombotic state. Meanwhile, we did not reveal any statistical differences between two groups in analysis of remaining secondary outcomes, including total adverse events, arrhythmia, AMI and cardiac arrest, acute mesenteric ischemia, ICU/hospital length of stay, and MV duration.

Several limitations should be taken into consideration when interpreting our findings. Firstly, the reported endpoints varied across included studies. Although the majority of trials provided 28-day or 30-day mortality, there were still a few studies that only presented data on ICU mortality, hospital mortality or 7-day mortality. However, we enrolled survival data of all included trials in the analysis of primary outcomes, which might potentially introduce bias. Given that, we performed sensitivity analysis and excluded studies that reported mortality in other phases. Correspondingly, the conclusions remained unchanged. Secondly, heterogeneity of some secondary endpoints was relatively high. We further conducted sensitivity analyses in order to unravel the source of heterogeneity. Thirdly, as the timing, duration, precise dose, delivery methods as well as weaning of vasopressin or its analogs were disparate and uncontrollable among included trials, it might render bias and affect the robustness of our conclusions. However, the detailed information was insufficient in many trials, which restricted us from performing further subgroup analyses. Finally, we failed to compare the clinical efficacy between vasopressin and its analogs. Thus, network meta-analyses were required to address this issue in the future.

## Conclusions

In this study, administration of vasopressin or its analogs showed no significant improvement in reducing 28-day or 30-day mortality among patients with septic shock when compared with those solely using catecholamines, while administration of terlipressin might be benefit for survival of septic shock patients comparing to catecholamines alone. Besides, septic shock patients receiving vasopressin or its analogs did present an increased risk of digital ischemia in comparison with those solely using catecholamines.

## Author Contributions

Y-MY, CR, and Z-FX conceived the meta-analysis. R-QY and D-MX extracted all data. L-XW, G-SW, Y-BZ, H-QZ, and QL undertook and refined the searches. R-QY, D-MX, and L-XW cowrote the paper. R-QY undertook the statistical analyses. All authors contributed to and revised the final manuscript.

## Funding

This work was supported by grants from the National Natural Science Foundation of China (Nos. 81730057, 81842025, 81801935, 81901957), the National Key Research and Development Program of China (No. 2017YFC1103302), and the Key Project of Military Medical Innovation Program of Chinese PLA (No. 18CXZ026).

## Conflict of Interest

The authors declare that the research was conducted in the absence of any commercial or financial relationships that could be construed as a potential conflict of interest.

## Abbreviations

AMI, acute myocardial infarction; CI, confidence intervals; GRADE, Grading of Recommendations, Assessment, Development and Evaluation; ICU, intensive care units; MAP, mean arterial pressure; MDs, mean differences; MeSH, Medical Subject Heading; NE, norepinephrine; PRISMA, Preferred Reporting Items for Systematic Reviews and Meta-Analysis; RCTs, randomized controlled trials; RRs, risk ratios.
