# Supplementary figures and images for: Clinical Efficiency of Vasopressin or Its Analogs in Comparison With Catecholamines Alone on Patients With Septic Shock: A Systematic Review and Meta-Analysis
Source: Front Pharmacol. 2020 May 6;11:563. doi: 10.3389/fphar.2020.00563 (PMC7218087; doi:10.3389/fphar.2020.00563)

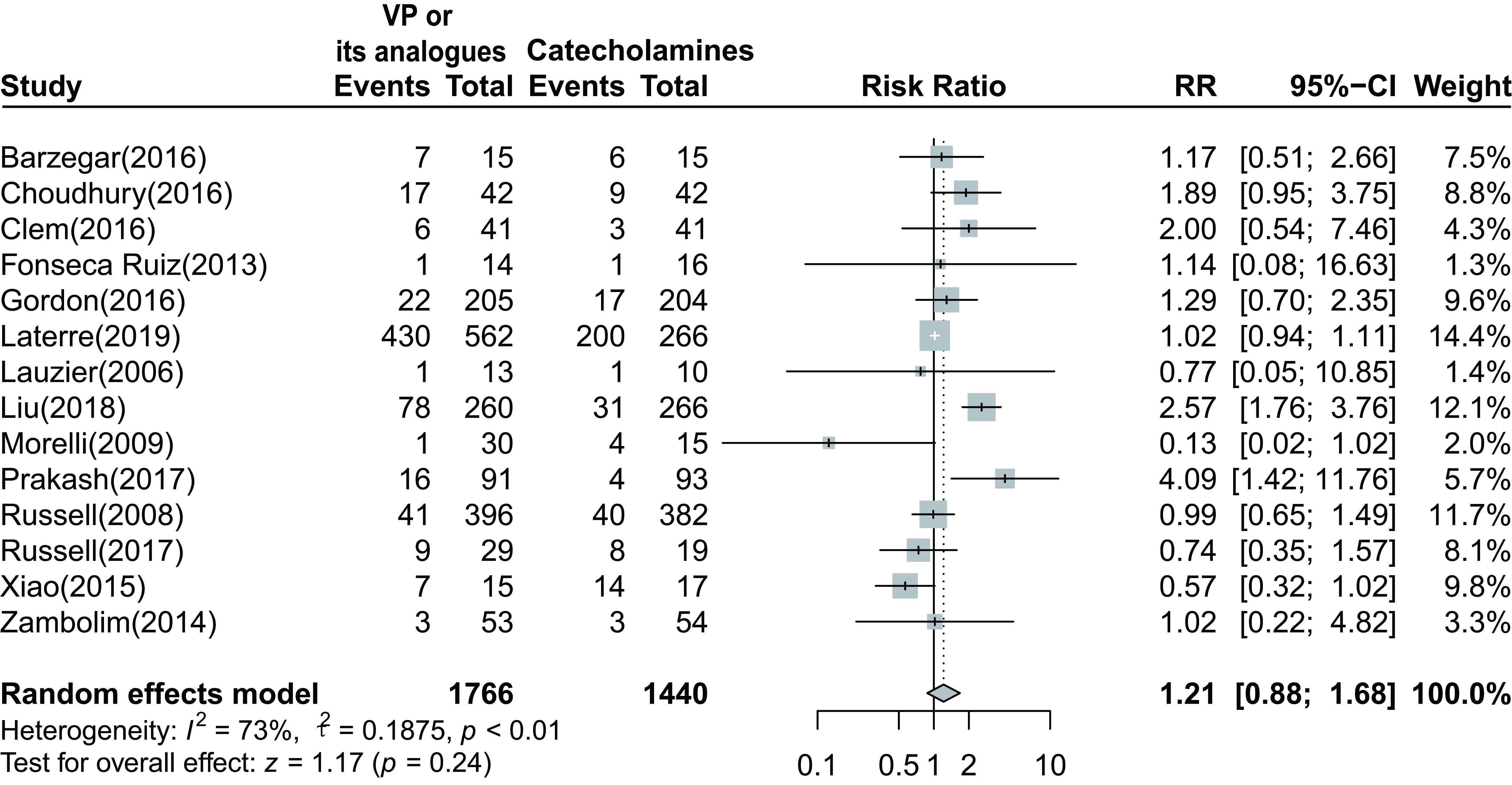

Supplement: Supplementary file 1 [file Image_1.tif]

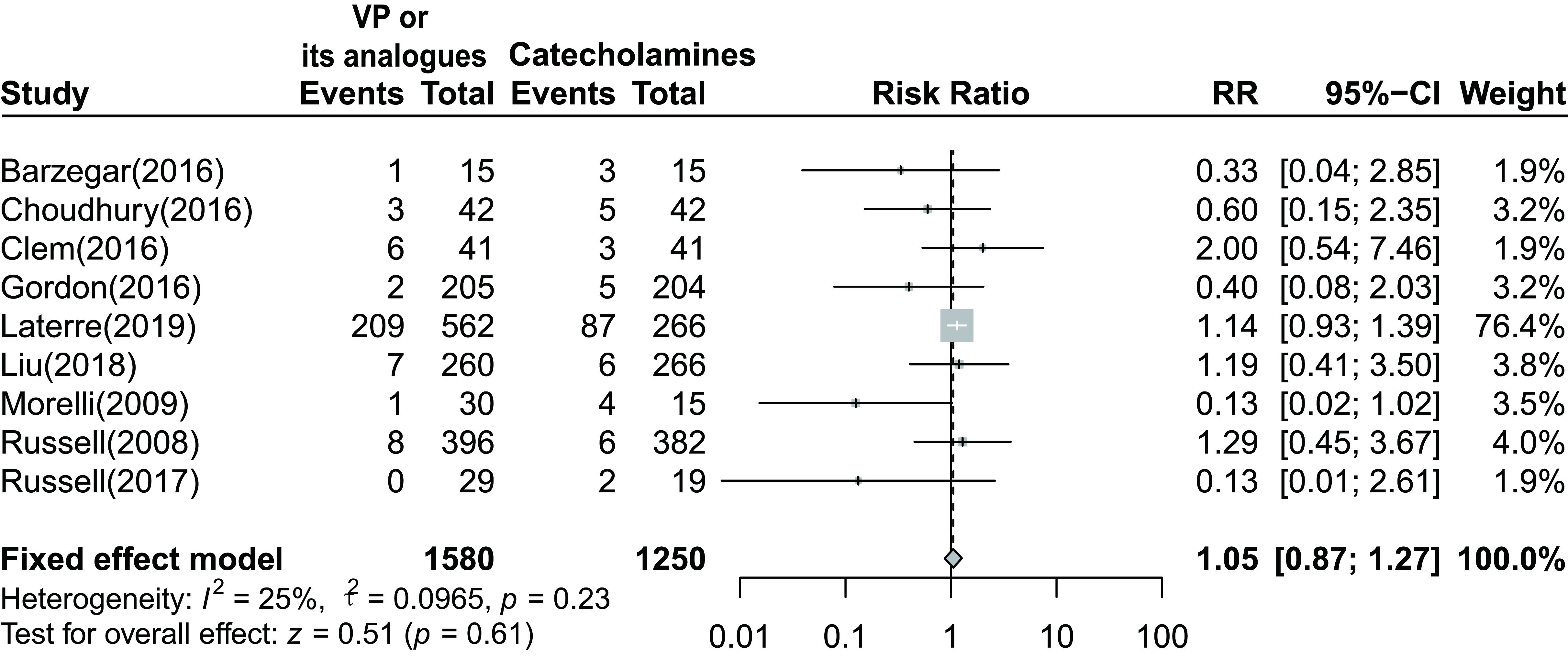

Supplement: Supplementary file 2 [file Image_2.tif]

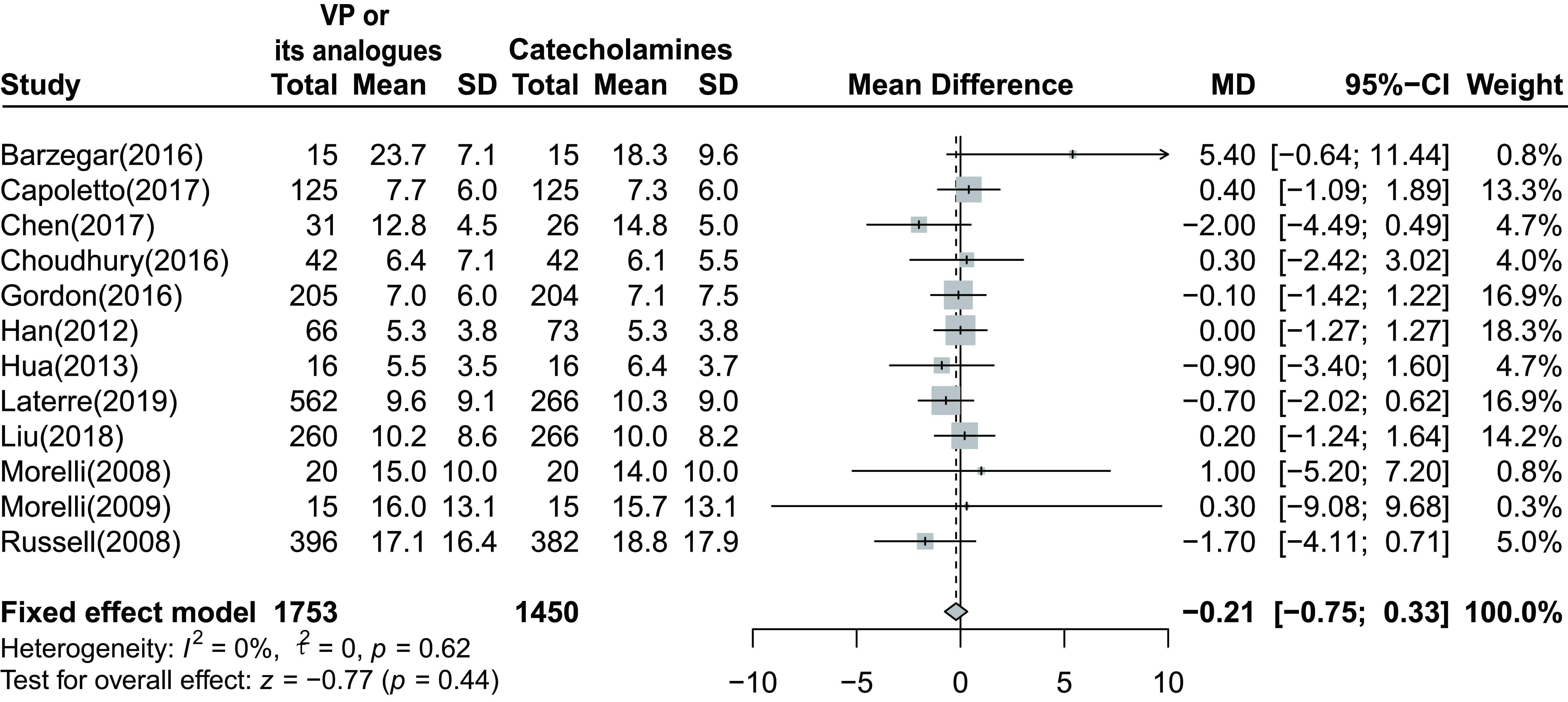

Supplement: Supplementary file 3 [file Image_3.tif]

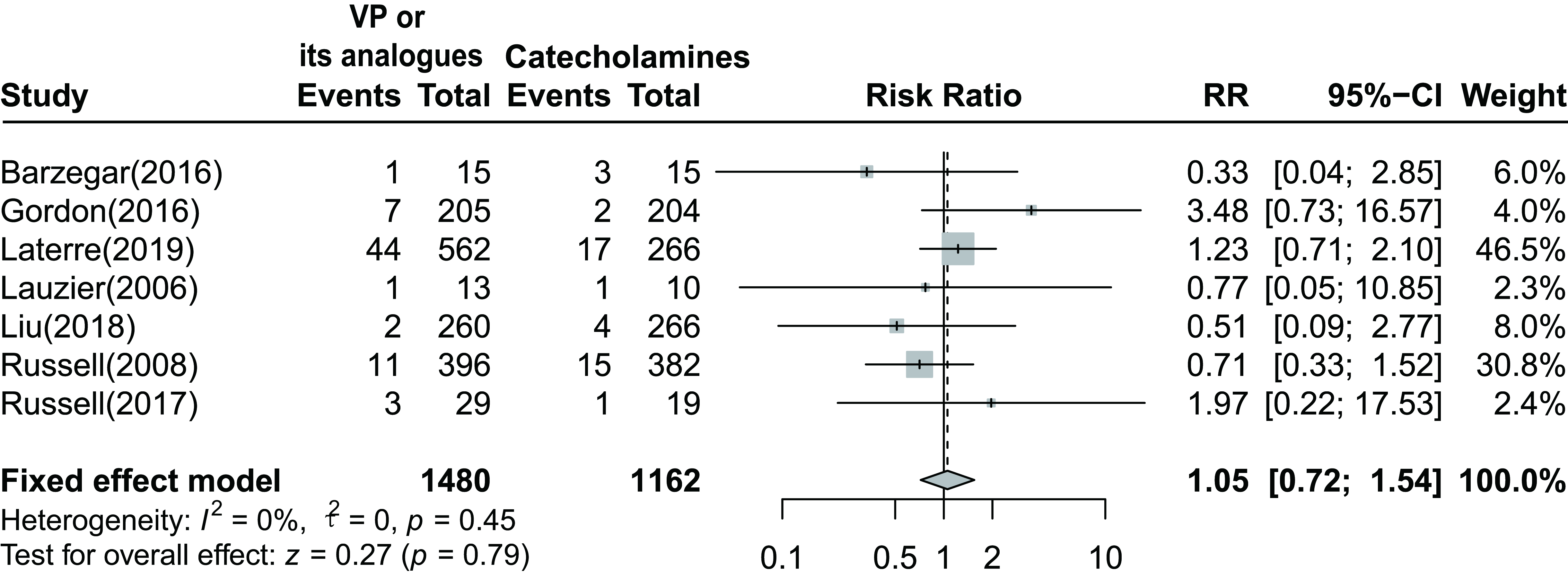

Supplement: Supplementary file 4 [file Image_4.tif]

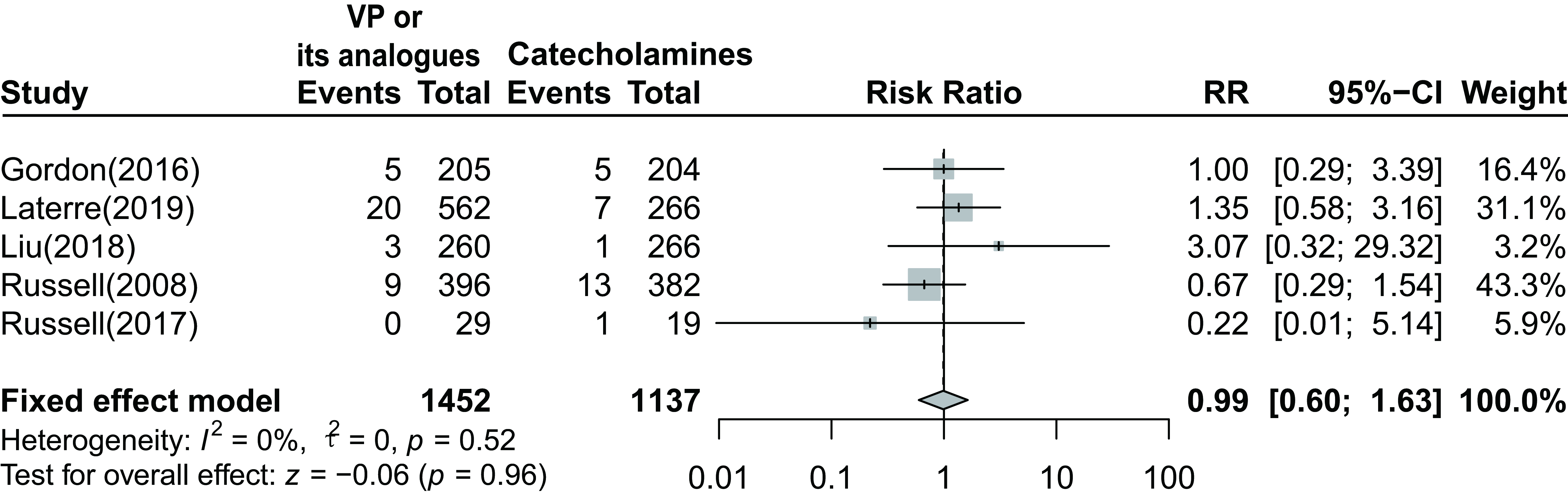

Supplement: Supplementary file 5 [file Image_5.tif]

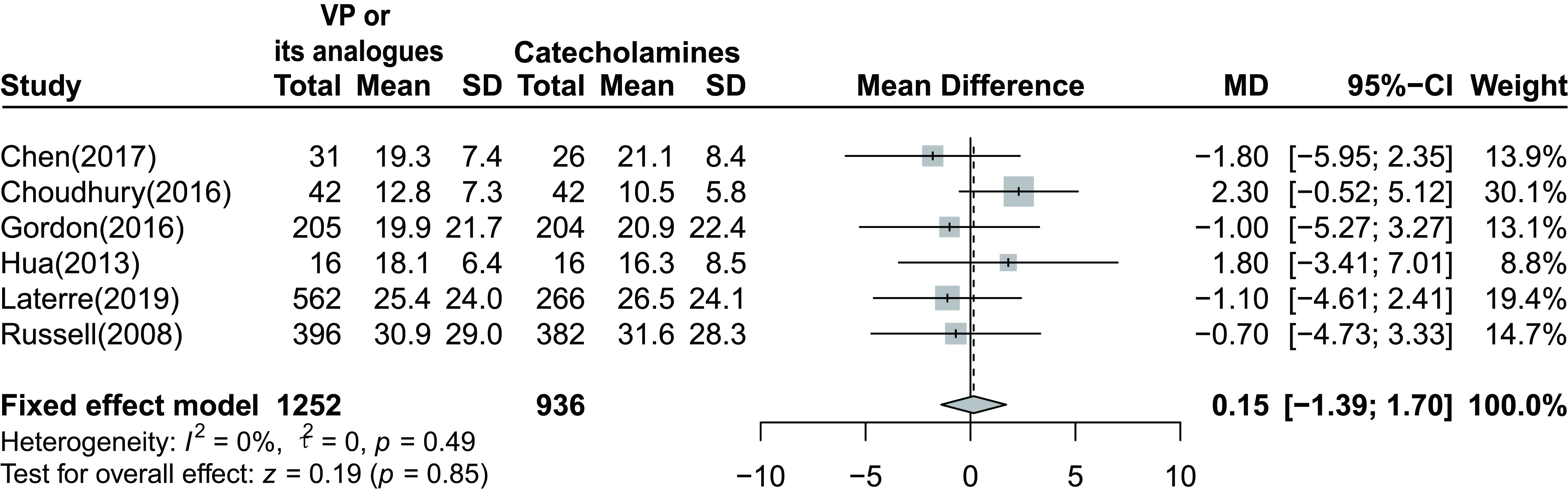

Supplement: Supplementary file 6 [file Image_6.tif]

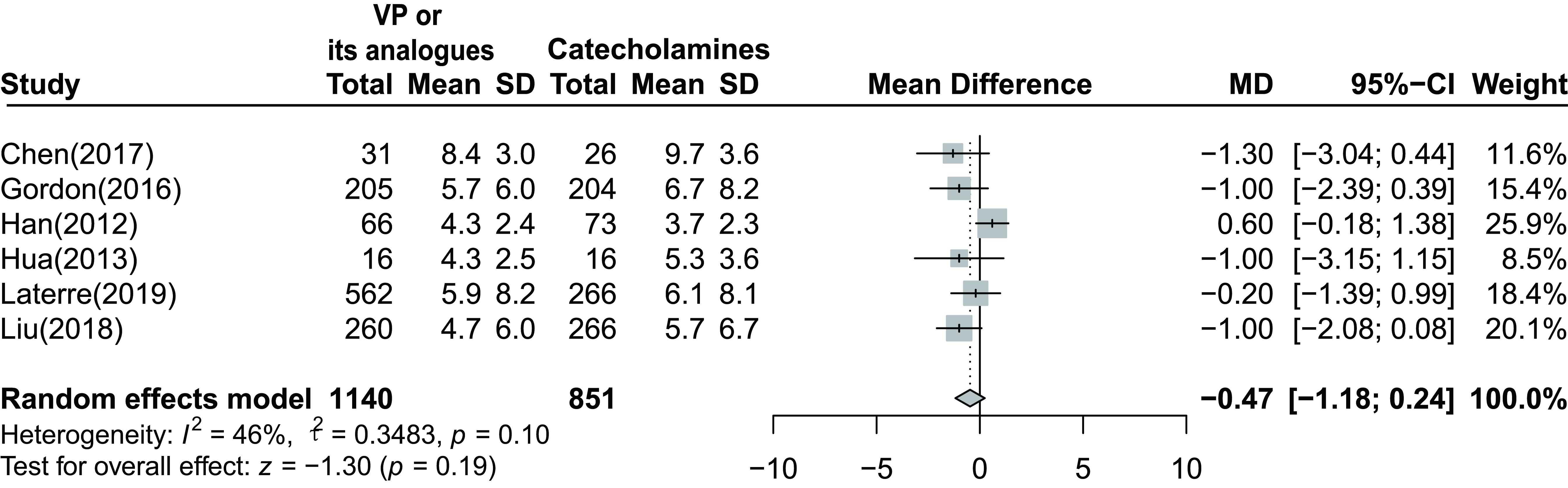

Supplement: Supplementary file 7 [file Image_7.tif]
