# Supplementary material for: Clinical Efficiency of Vasopressin or Its Analogs in Comparison With Catecholamines Alone on Patients With Septic Shock: A Systematic Review and Meta-Analysis
Source: Front Pharmacol. 2020 May 6;11:563. doi: 10.3389/fphar.2020.00563 (PMC7218087; doi:10.3389/fphar.2020.00563)
Supplement: Supplementary file 9 [file Table_1.docx]

**Supplemental Table S1. Jadad scale of randomized controlled trials included in the meta-analysis.**

| **Study** | **Randomization** | **The method of randomization was described and appropriate** | **The study was described as randomized** | **Double- blinding** | **The method of double blinding was described and appropriate** | **The study was described as double blind** | **Withdrawals and dropouts** | **Total Jadad score** |  |
| --- | --- | --- | --- | --- | --- | --- | --- | --- | --- |
|  |  |  |  |  |  |  |  |  |  |
|  |  |  |  |  |  |  |  |  |  |
|  |  |  |  |  |  |  |  |  |  |
| Acevedo et al | Yes | No | Yes | No | No | No | No | 1 |  |
| Albanese et al | Yes | Yes | No | No | No | No | Yes | 2 |  |
| Barzegar et al | Yes | Yes | No | Yes | No | Yes | Yes | 4 |  |
| Capoletto et al | Yes | Yes | No | Yes | No | Yes | No | 3 |  |
| Chen et al | Yes | No | Yes | Yes | No | Yes | Yes | 3 |  |
| Choudhury et al | Yes | Yes | No | Yes | No | Yes | Yes | 4 |  |
| Clem et al | Yes | No | Yes | No | No | No | No | 1 |  |
| Fonseca Ruiz et al | Yes | Yes | No | Yes | No | Yes | Yes | 4 |  |
| Gordon et al | Yes | Yes | No | Yes | Yes | No | Yes | 5 |  |
| Han et al | Yes | No | Yes | No | No | No | No | 1 |  |
| Hua et al | Yes | Yes | No | No | No | No | No | 2 |  |
| Laterre et al | Yes | Yes | No | Yes | Yes | No | Yes | 5 |  |
| Lauzier et al | Yes | Yes | No | No | No | No | Yes | 3 |  |
| Liu et al | Yes | Yes | No | Yes | Yes | Yes | Yes | 5 |  |
| Morelli et al (2008) | Yes | Yes | No | No | No | No | Yes | 3 |  |
| Morelli et al (2009) | Yes | Yes | No | No | No | No | Yes | 3 | |
| Oliveira et al | Yes | No | Yes | Yes | No | Yes | Yes | 3 | |
| Prakash et al | Yes | No | Yes | No | No | No | No | 1 | |
| Russell et al (2008) | Yes | Yes | No | Yes | Yes | No | Yes | 5 | |
| Russell et al (2017) | Yes | Yes | No | Yes | Yes | No | Yes | 5 | |
| Svoboda et al | Yes | Yes | No | No | No | No | Yes | 3 | |
| Xiao et al | Yes | Yes | No | Yes | No | Yes | No | 3 | |
| Zambolim et al | Yes | No | Yes | No | No | No | No | 1 | |
