# Supplementary material for: Clinical Efficiency of Vasopressin or Its Analogs in Comparison With Catecholamines Alone on Patients With Septic Shock: A Systematic Review and Meta-Analysis
Source: Front Pharmacol. 2020 May 6;11:563. doi: 10.3389/fphar.2020.00563 (PMC7218087; doi:10.3389/fphar.2020.00563)
Supplement: Supplementary file 10 [file Table_2.docx]

**Supplemental Table S2. Summary of findings**

| **Outcomes** | **Illustrative comparative risks* (95% CI)** | | **Relative effect (95% CI)** | **No of Participants (studies)** | **Quality of the evidence (GRADE)** | **Comments** |
| --- | --- | --- | --- | --- | --- | --- |
|  | Assumed risk | Corresponding risk |  |  |  |  |
|  | **catecholamine** | **Vasopressin or its analogue** |  |  |  |  |
| **28-day or 30-day Mortality** | **Study population** | | **RR 0.94**  (0.87 to 1.01) | 4225 (23 studies) |  |  |
|  | **426 per 1000** | **400 per 1000** (370 to 430) |  |  |  |  |
|  | **Moderate** | |  |  |  |  |
|  | **466 per 1000** | **438 per 1000** (405 to 471) |  |  |  |  |
| **Total adverse events** | **Study population** | | **RR 1.21**  (0.88 to 1.68) | 3206 (14 studies) |  |  |
|  | **237 per 1000** | **287 per 1000** (208 to 398) |  |  |  |  |
|  | **Moderate** | |  |  |  |  |
|  | **111 per 1000** | **134 per 1000** (98 to 186) |  |  |  |  |
| **Arrhythmia** | **Study population** | | **RR 1.05**  (0.87 to 1.27) | 2830 (9 studies) |  |  |
|  | **97 per 1000** | **102 per 1000** (84 to 123) |  |  |  |  |
|  | **Moderate** | |  |  |  |  |
|  | **105 per 1000** | **110 per 1000** (91 to 133) |  |  |  |  |
| **AMI and cardiac arrest** | **Study population** | | **RR 1.05**  (0.72 to 1.54) | 2642 (7 studies) |  |  |
|  | **37 per 1000** | **39 per 1000** (27 to 57) |  |  |  |  |
|  | **Moderate** | |  |  |  |  |
|  | **53 per 1000** | **56 per 1000** (38 to 82) |  |  |  |  |
| **Digital ischemia** | **Study population** | | **RR 2.65**  (1.26 to 5.56) | 2929 (9 studies) |  |  |
|  | **16 per 1000** | **42 per 1000** (20 to 89) |  |  |  |  |
|  | **Moderate** | |  |  |  |  |
|  | **15 per 1000** | **40 per 1000** (19 to 83) |  |  |  |  |
| **Acute mesenteric ischemia** | **Study population** | | **RR 0.99**  (0.6 to 1.63) | 2589 (5 studies) |  |  |
|  | **24 per 1000** | **24 per 1000** (14 to 39) |  |  |  |  |
|  | **Moderate** | |  |  |  |  |
|  | **26 per 1000** | **26 per 1000** (16 to 42) |  |  |  |  |
| **ICU length of stay** |  | The mean icu length of stay in the intervention groups was **0.21 lower** (0.75 lower to 0.33 higher) |  | 3203 (12 studies) |  |  |
| **Hospital length of stay** |  | The mean hospital length of stay in the intervention groups was **0.15 higher** (1.39 lower to 1.7 higher) |  | 2188 (6 studies) |  |  |
| **MV duration** |  | The mean mv duration in the intervention groups was **0.47 lower** (1.18 lower to 0.24 higher) |  | 1991 (6 studies) |  |  |
| *The basis for the **assumed risk** (e.g. the median control group risk across studies) is provided in footnotes. The **corresponding risk** (and its 95% confidence interval) is based on the assumed risk in the comparison group and the **relative effect** of the intervention (and its 95% CI).  **CI:** Confidence interval; **RR:** Risk ratio; | | | | | | |
| GRADE Working Group grades of evidence **High quality:** Further research is very unlikely to change our confidence in the estimate of effect.  **Moderate quality:** Further research is likely to have an important impact on our confidence in the estimate of effect and may change the estimate. **Low quality:** Further research is very likely to have an important impact on our confidence in the estimate of effect and is likely to change the estimate. **Very low quality:** We are very uncertain about the estimate. | | | | | | |
|  |  |  |  |  |  |  |
