# Supplementary material for: Clinical Efficiency of Vasopressin or Its Analogs in Comparison With Catecholamines Alone on Patients With Septic Shock: A Systematic Review and Meta-Analysis
Source: Front Pharmacol. 2020 May 6;11:563. doi: 10.3389/fphar.2020.00563 (PMC7218087; doi:10.3389/fphar.2020.00563)
Supplement: Supplementary file 11 [file DataSheet_1.docx]

**Figure Legends**

**Supplemental Figure S1.** Forest plot of total adverse events comparing vasopressin or its analogues to catecholamines alone among septic shock patients. VP, vasopressin; RR, risk ratio; CI, confidence interval.

**Supplemental Figure S2.** Forest plot of arrhythmia comparing vasopressin or its analogues to catecholamines alone among septic shock patients. VP, vasopressin; RR, risk ratio; CI, confidence interval.

**Supplemental Figure S3.** Forest plot of ICU length of stay comparing vasopressin or its analogues to catecholamines alone among septic shock patients. VP, vasopressin; MD, mean difference; CI, confidence interval.

**Supplemental Figure S4.** Forest plot of AMI and cardiac arrest comparing vasopressin or its analogues to catecholamines alone among septic shock patients. VP, vasopressin; RR, risk ratio; CI, confidence interval.

**Supplemental Figure S5.** Forest plot of acute mesenteric ischemia comparing vasopressin or its analogues to catecholamines alone among septic shock patients. VP, vasopressin; RR, risk ratio; CI, confidence interval.

**Supplemental Figure S6.** Forest plot of hospital length of stay comparing vasopressin or its analogues to catecholamines alone among septic shock patients. VP, vasopressin; MD, mean difference; CI, confidence interval.

**Supplemental Figure S7.** Forest plot of MV duration comparing vasopressin or its analogues to catecholamines alone among septic shock patients. VP, vasopressin; MD, mean difference; CI, confidence interval.

**Supplemental Figure S8.** Funnel plot regarding to 28-day or 30-day mortality.
